# Research and data sovereignty in genetically admixed populations

**DOI:** 10.1007/s00125-025-06383-w

**Published:** 2025-03-11

**Authors:** Linda Zollner, Rajiv Kumar, Justo Lorenzo Bermejo

**Affiliations:** 1 Statistical Genetics Research Group, Institute of Medical Biometry, Heidelberg University, Heidelberg, Germany; 2 Division of Proteomics of Stem Cells and Cancer, German Cancer Research Center, Heidelberg, Germany; 3 Laboratory of Biostatistics for Precision Oncology, Institut de cancérologie Strasbourg Europe, Strasbourg, France

**Keywords:** Data sovereignty, Disease predisposition, Genetic admixture

*To the Editor:* We appreciate the recommendations in the article ‘Scientists and scientific journals should adhere to ethical standards for the use and reporting of data from Indigenous people’, recently published in *Diabetologia* [1]. We fully agree with the authors about the importance of adopting international standards on Indigenous data sovereignty and ethical use of Indigenous samples. They cite the United Nations Declaration on the Rights of Indigenous Peoples, which gives them the right to ‘maintain, control, protect and develop their cultural heritage, traditional knowledge and traditional cultural expressions, as well as the manifestations of their sciences, technologies and cultures, including human and genetic resources’ [2]. They rightly argue that some scientists working with biological and genetic data from Indigenous people still do not respect these rights, and do not sufficiently involve Indigenous communities in research, which hinders Indigenous peoples’ participation in future studies as well as scientific discoveries.

However, we strongly refute the authors’ reference to our recent publication as an example of research that does not meet ethical standards [3]. The citation is first inappropriate, because our results came not from Indigenous people but from the general Chilean population. The genome of modern Chileans is the result of genetic admixture between two major Indigenous peoples, the Mapuche in southern Chile and the Aymara in the north; the Spaniards who arrived in Chile in the mid-16^th^ century; the enslaved African people who arrived in the 17^th^ century; and later migrations [4]. The data investigated were collected as part of the European–Latin American Consortium towards Eradication of Preventable Gallbladder Cancer (EULAT Eradicate GBC). After a thorough explanation process, which included answering all questions from potential participants in lay language, study participants gave written consent for using their samples and data, of which they are the sovereign owners, for our study [3].

The reference to our recent publication is also incomplete, because the authors make no mention of our comprehensive activities to protect the rights of Indigenous peoples, described in detail in the ‘Patient and public involvement’ section of the article [3]. Briefly, the dissemination videos of the project are available in three Indigenous languages: Mapudungun, Quechua and Aymara. Although our study did not directly involve Indigenous people, the manuscript was reviewed by an expert on Mapuche culture from a Chilean university. We have also recently organised two events on this very topic: a symposium on ‘Indigenous American Ancestry in Genetic Research’ at the joint meeting of the Chilean Genetics Society and the Chilean Society of Evolution, and the international summer school ‘Ancestry Meets Molecular Health’ to discuss and improve ways to involve Indigenous communities in the design and implementation of research projects.

Latin Americans have a higher risk than Europeans of developing gallbladder cancer, an aggressive tumour that mainly affects women. In our study, we investigated whether the risk difference is related to confounding factors (e.g. worse access to healthcare for Chileans with high proportions of Indigenous ancestry) or whether there is an unconfounded relationship between genetic Indigenous ancestry and gallbladder cancer risk. Answering this question has important implications for cancer prevention.

We respectfully request that authors and readers of *Diabetologia* be made aware of our clarifications. Inappropriate citations and misrepresentations, even if unintentional, can mislead stakeholders and potentially affect the impact of our research, which aims to improve health in the region.

## References

[1] Yracheta J, Morriseau T, Dale K, Gerth A, McGavock J (2024) Scientists and scientific journals should adhere to ethical standards for the use and reporting of data from indigenous people. Diabetologia 67(11):2404–2407. 10.1007/s00125-024-06236-y 39103722 10.1007/s00125-024-06236-y

[2] United Nations (2018) United Nations Declaration on the Rights of Indigenous Peoples. Available from: https://www.un.org/development/desa/indigenouspeoples/declaration-on-%20the-rights-of-indigenous-peoples.html. Accessed 3 Feb 2025

[3] Zollner L, Boekstegers F, Barahona Ponce C et al (2023) Gallbladder cancer risk and indigenous south american mapuche ancestry: instrumental variable analysis using ancestry-informative markers. Cancers (Basel) 15(16):4033. 10.3390/cancers15164033 37627062 10.3390/cancers15164033 PMC10452561

[4] Ruiz-Linares A, Adhikari K, Acuña-Alonzo V et al (2014) Admixture in Latin America: geographic structure, phenotypic diversity and self-perception of ancestry based on 7,342 individuals. PLoS Genet 10(9):e1004572. 10.1371/journal.pgen.1004572 25254375 10.1371/journal.pgen.1004572 PMC4177621

